# 140. Follow-up Blood Cultures for Gram-Negative Bacteremia among Adults with Cancer

**DOI:** 10.1093/ofid/ofad500.213

**Published:** 2023-11-27

**Authors:** David Haak, Karen Fong, Emily S Spivak, Hannah Imlay

**Affiliations:** University of Utah, Salt Lake City, Utah; University of Utah Health, Salt Lake City, Utah; University of Utah School of Medicine, Salt Lake City, UT; University of Utah Health, Salt Lake City, Utah

## Abstract

**Background:**

Current evidence suggests that performing follow up blood cultures (FUBC) in patients with Gram-negative bacteremia (GNB) may not improve outcomes and can lead to unnecessary healthcare expenditure. However, studies often exclude more fragile patient populations, such as those with active malignancy. In a cohort of patients with active malignancy and GNB, we aimed to quantify the rate of performing FUBCs, the rate of positivity, and to compare characteristics of patients with and without positive FUBCs.

**Methods:**

Patients admitted to University of Utah Health with active malignancy and GNB during 2019 were identified. Manual chart review was performed to collect demographics, clinical characteristics, management and outcome measures. Patients who had positive FUBC were compared to those with negative FUBC. Patients who died or went to comfort care only within 48h of admission were excluded.

**Results:**

56 patients were identified. FUBCs were performed in 51 patients (70 FUBCs total) during the 4 days after initial positive blood culture. 4 FUBCs in 4 patients were positive. Of the positive FUBC, 2 (50%) were collected within 24 hours of the initial blood culture (Figure 1). 1 patient with a positive FUBC (25%) had not had source control achieved (Table 1).

90-day readmission (50% vs 34%) and recurrent GNB with the same organism (25% vs 8.5%) were higher in the positive FUBC group compared to the negative FUBC group. 90-day mortality (25% vs 28%) was similar between the two groups (Table 1).

**Figure:**
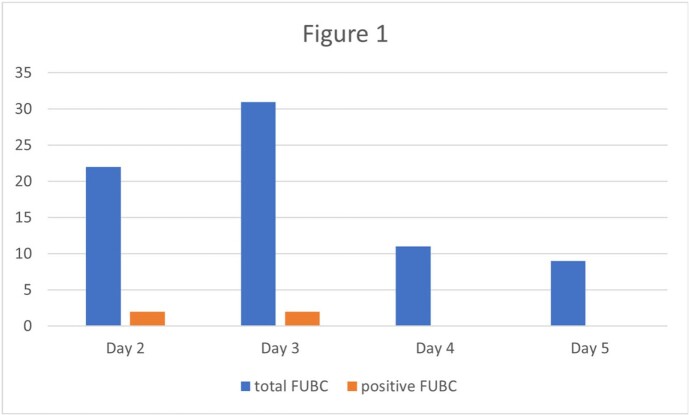

**Figure:**
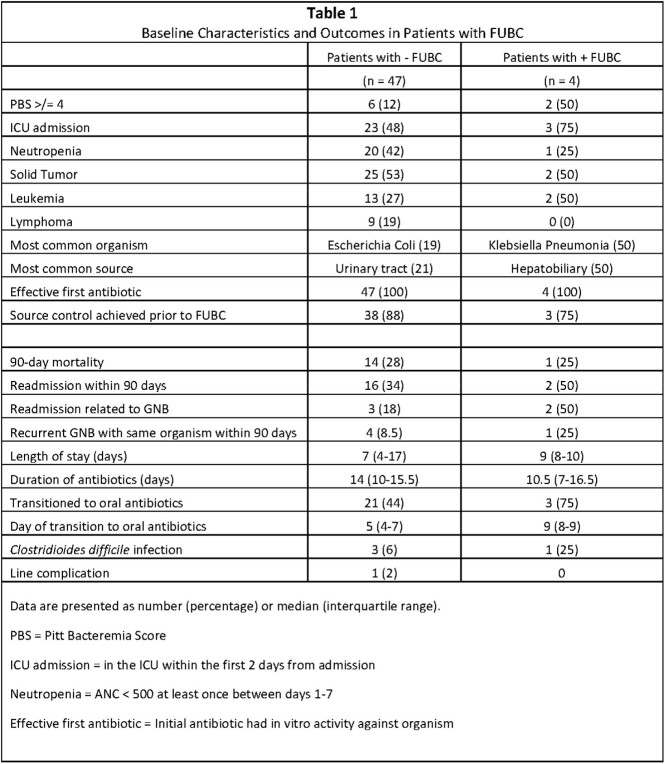

**Conclusion:**

In hospitalized patients with active malignancy and GNB, FUBC were performed frequently but the majority were negative. Of the positive FUBC, a significant proportion were either collected within 24 hours of starting active antimicrobials, limiting their significance. Prospective studies of standardized blood culture collection are needed to characterize the natural history of GNB and define when positive FUBC are clinically meaningful. These data suggest use of FUBC use could be reduced by limiting use to more than 24 hours after both appropriate antibiotic therapy is initiated and source control is achieved.

**Disclosures:**

**All Authors**: No reported disclosures

